# Light-sheets and smart microscopy, an exciting future is dawning

**DOI:** 10.1038/s42003-023-04857-4

**Published:** 2023-05-09

**Authors:** Stephan Daetwyler, Reto Paul Fiolka

**Affiliations:** 1 Lyda Hill Department of Bioinformatics, University of Texas Southwestern Medical Center, Dallas, TX USA; 2 Department of Cell Biology, University of Texas Southwestern Medical Center, Dallas, TX USA

**Keywords:** Microscopy, Bioinformatics, Biological fluorescence

## Abstract

Light-sheet fluorescence microscopy has transformed our ability to visualize and quantitatively measure biological processes rapidly and over long time periods. In this review, we discuss current and future developments in light-sheet fluorescence microscopy that we expect to further expand its capabilities. This includes smart and adaptive imaging schemes to overcome traditional imaging trade-offs, i.e., spatiotemporal resolution, field of view and sample health. In smart microscopy, a microscope will autonomously decide where, when, what and how to image. We further assess how image restoration techniques provide avenues to overcome these tradeoffs and how “open top” light-sheet microscopes may enable multi-modal imaging with high throughput. As such, we predict that light-sheet microscopy will fulfill an important role in biomedical and clinical imaging in the future.

## Abstract (web summary)

In this review, the future of fluorescence imaging with light sheets is discussed, together with the advent of adaptive and smart imaging schemes.

## Introduction

**Fig. 1:**
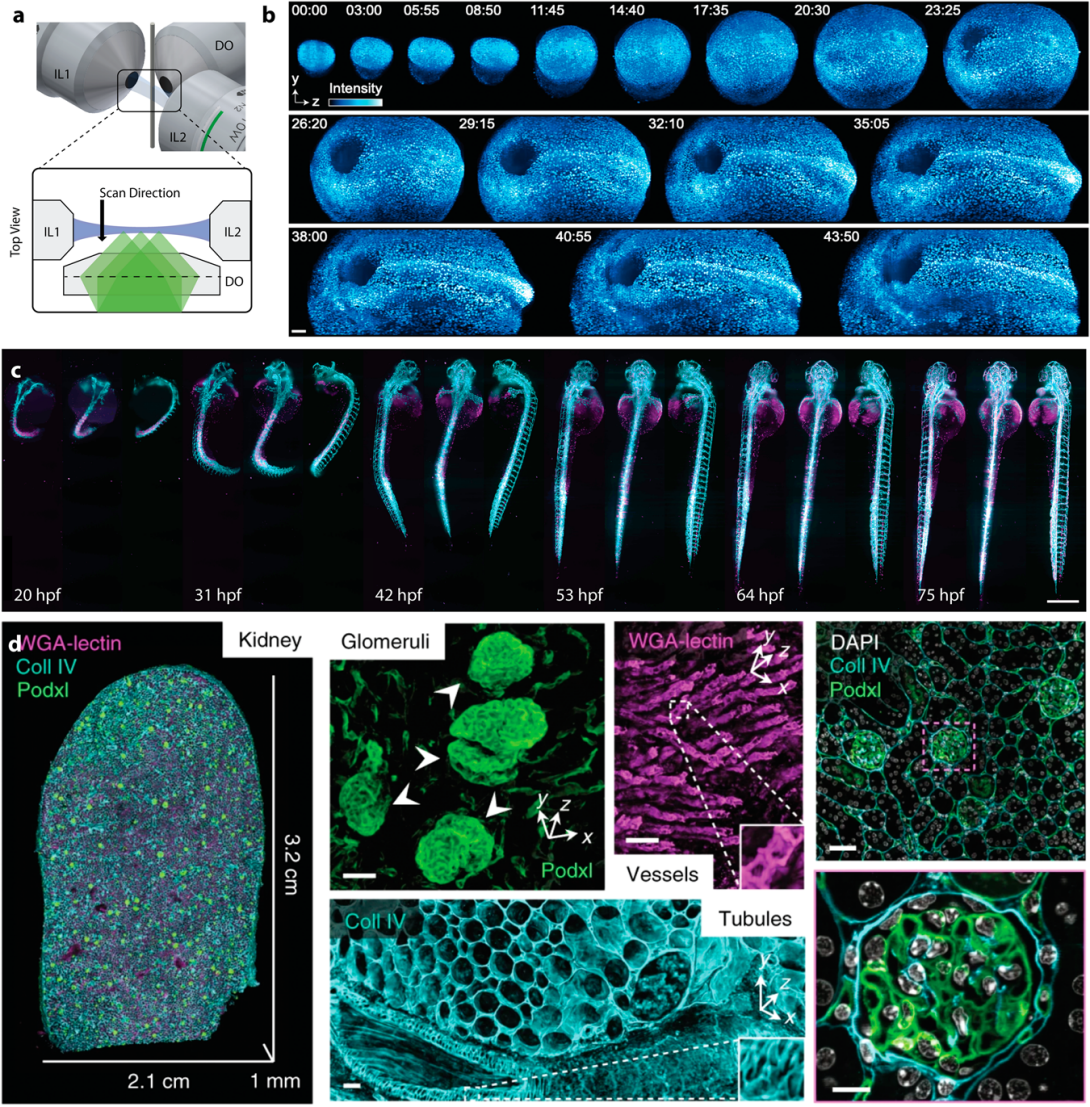
Light-sheet microscopy provides fast imaging with minimized photo-toxicity and photo-bleaching, enabling diverse applications from imaging developmental processes to imaging of large, cleared tissues. **a** Traditional light-sheet microscopy such as three-objective Selective Plane Illumination Microscopy (SPIM) relies on an orthogonal arrangement of the illumination (blue; illumination objectives IL1 and IL2) and detection (green; detection objective DO). This ensures that the axial resolution of imaging is mainly governed by the thickness of the light-sheet (blue) enabling imaging across large samples with widefield detection (green) and good optical sectioning. To acquire a 3D volume, the sample is scanned along the detection axis either by moving the sample itself or by scanning the light sheet together with the objective in the detection path. **b**–**d** Prime examples of imaging with light-sheets include continuous, long-term imaging of developmental processes in mouse and zebrafish embryos, and imaging of cleared tissue with subcellular resolution. **b** Katie McDole et al.^4^ characterized the cellular movements involved in mouse development from early streak (E6.5) to somite stages (E8.5) by imaging a CAGTAG1 expressing mouse embryo with a histone marker (H2B-eGFP) for over 44 h. Scale bar: 100 μm. **c** Selected projections from multi-view imaging^5^ (three angles) of the growing embryonic zebrafish vasculature labeled with the fluorescent vascular marker (*Tg(kdrl:EGFP)*, cyan) and the red blood cell marker (*Tg(GATA1a:dsRed), magenta*), imaged from 20 h post-fertilization (hpf) to 86 hpf. Scale bar: 500 μm **d** Adam Glaser et al.^37^ performed large-scale imaging of an expanded slice of kidney of 3.2 cm × 2.1 cm size and 1 mm thickness. High-resolution regions of interest revealed the morphology of glomeruli (Scale bar: 40 μm), vessels (Scale bar: 80 μm) and tubules (Scale bar: 50 μm). The increased resolution due to expansion was further demonstrated with a multi-channel zoom-in of DAPI-counterstained tissue (Scale bars: 100 μm [top] and 20 μm [bottom]). The scale bars thereby indicate the dimensions of the native unexpanded tissue. Panel **b** was adapted with permission from Katie McDole et al. (2018)^4^. Panel **c** adapted from Daetwyler et al. (2019)^5^. Panel **d** adapted from Glaser et al. (2019)^37^.

Over the past decades, microscopes have provided us with invaluable insights on how biological processes are organized in space and time. A core innovation has been the selective labeling of proteins and lipids with fluorescent markers^1,2^, enabling fluorescent microscopy techniques such as light-sheet fluorescence microscopy (or light-sheet microscopy for short)^3^. Light-sheet microscopy enables us to visualize, quantify and dynamically track structural components in vivo^4–6^ and in vitro^7–9^. The fundamentals of light-sheet microscopy are covered in several reviews^10–19^, but in short, it relies on an orthogonal separation of the illumination and detection path, enabling selective illumination of a whole imaging plane and simultaneous widefield detection (Fig. 1a).

In this review, we will look ahead and discuss potential future avenues of fluorescence imaging with light-sheets. We will describe how volumetric and temporal imaging barriers limit the application of optical microscopy to capture large specimens with high spatiotemporal resolution and explore strategies to overcome them. This includes progress in technology, novel smart and adaptive imaging schemes and image restoration techniques. Moreover, we will review how such schemes will go hand-in-hand with flexible, open top light-sheet microscopes to enable multi-modality imaging with high throughput.

## The core of light-sheet fluorescence microscopy

Light-sheet microscopy stands out for its efficient and gentle 3D imaging capacity. It is characterized by a light intensity distribution in the shape of a sheet, which illuminates the focal plane of a microscope detection system (Fig. 1a)^3,16^. This provides many advantages. Most importantly, only (or at least predominantly) the focal plane of the detection system is illuminated, which results in optical sectioning and minimal out-of-focus excitation^15,16,19^. This leads to crisp images devoid of blur, and massively reduced sample bleaching compared to conventional, epi-fluorescent microscopy techniques, such as widefield or confocal^10,20^.

The excitation of the focal plane is traditionally achieved with one or two illumination objectives to launch the light-sheet(s)^3,21^. Thereby, coherent laser light is shaped into Gaussian^3^, top hat^21^, single or multiple Bessel^22,23^, Airy^24^ or other^25,26^ beams to create an intensity distribution in the sample that approximates a sheet over a finite distance. Volumetric imaging is achieved by either scanning the sample^3^, increasing the depth of focus^27^, or moving the sheet and the detection^28,29^.

Detection of the excited fluorophores is achieved by capturing the fluorescence signal of the illuminated plane with widefield detection on a scientific camera^16^. To understand the benefit of light-sheet microscopy, the concept of spatial duty cycle is important. It describes how long a fluorophore is on during the duration of an exposure. As the entire plane is illuminated, the spatial duty cycle is much higher with light-sheet microscopy compared to conventional point-scanning confocal microscopes, where only a fraction of the volume is scanned at once. Consequently, lower laser powers can be applied to produce a similar signal. This is important as many photo-bleaching and photo-toxic effects are highly nonlinear to the excitation intensity^10,20^. However, the widefield detection limits the optical penetration depth of light-sheet microscopes, as scattering occludes imaging deeper in tissues. Nevertheless, for small organoids and model organisms, light-sheet microscopy can be applied, especially when combined with image fusion^30^, which combines information from different orientations of the sample. Thereby, areas that would otherwise be occluded by scattering can be visited from their most favorable viewing angle^15,31,32^. Multiple views can be acquired by sample rotation, or in recent implementations, up to four objectives provide optical paths for alternating between light-sheet illumination and detection^33–35^.

These abilities have allowed light-sheet microscopes to gently acquire 3D data over hundreds to thousands of stacks per sample. The resulting data revealed and enabled quantification of dynamic processes such as subcellular signaling and morphology^23,25^, embryogenesis over durations of days^4,5,36^ (Fig. 1b, c) and provided fast acquisition of large cleared tissues with subcellular resolution^37–40^ (Fig. 1d).

## Limitations of current imaging systems

**Fig. 2:**
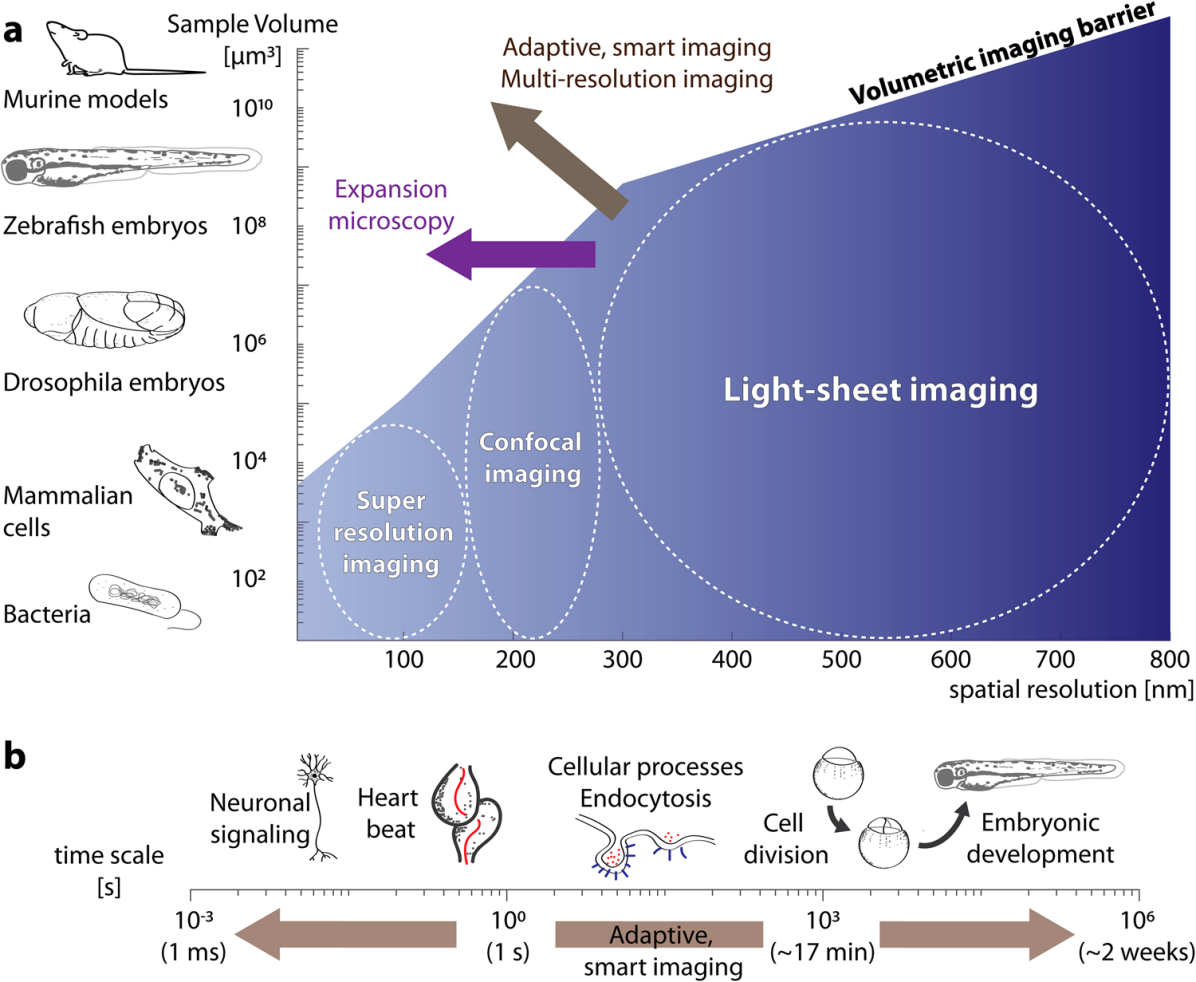
Expansion microscopy, and novel adaptive, smart imaging methods combined with multi-resolution imaging will expand the available imaging capabilities. **a** Current imaging techniques such as light-sheet, confocal and super-resolution microscopy are limited in the volume they can image due to technical and practical limitations (blue gradient: from low to high spatial resolution; white dashed circles indicate the predominant application regimes of the three microscopy techniques). To overcome this volumetric imaging barrier, expansion microscopy enables lower resolution imaging techniques to acquire with an effectively higher resolution. Additionally, we expect novel adaptive, smart imaging techniques and multi-resolution imaging to overcome the volumetric imaging barrier by selectively imaging parts of a large volume with high-resolution. Moreover, image reconstruction algorithms, such as compressed sensing and deep learning approaches, will provide avenues to obtain high-resolution images from partially sampled, large volumes **b** Additionally, we expect that adaptive and smart imaging schemes will overcome the temporal imaging barrier to image fast processes selectively over long time periods.

Despite the fast and gentle volumetric imaging provided by light-sheet and other fluorescence microscopes^16^, they are ultimately constrained by volumetric and temporal imaging barriers (Fig. 2). While the former refers to our inability to image large specimens with high resolution, the latter refers to our inability to image very fast processes continuously over extended time periods.

The volumetric imaging barrier (Fig. 2a) is given by the maximum volumetric reach of a given imaging technology. For example, large specimens, such as a whole mouse, cannot currently be imaged with confocal or super-resolution imaging in their entirety. This is in part governed by physical limitations, such as optical penetration due to light-scattering, and optical engineering, e.g. a trade-off between Numerical Aperture and working distance as well as field of view^41^.

Additionally, proper Nyquist sampling can become rate limiting so that imaging a large specimen at high resolution is impractical. To illustrate this, a common voxel dwell time for a conventional laser scanning confocal microscope with 250 nm resolution is ~1 μs^42^, and thus it would take ~4.2 s to capture a 2048 × 2048 × 1 confocal image. Doubling the resolution with an Airyscan microscope to 120 nm^43^, would require for the same field of view ~16.8 s. Imaging a Drosophila egg^44^ of size 9 × 10^−3^ mm^3^ (0.18 mm width, 0.51 mm length) using a confocal microscope with Nyquist sampling would thus take 76 min, or over 10 h with an Airyscan confocal. This prevents imaging at rates enabling studies of cellular dynamics, such as endocytosis processes that occur within a minute^45^. While light-sheet microscopy is much faster due to its widefield acquisition and high duty cycle, high-resolution versions of it such as Lattice light-sheet microscopy^23,46^ or Axially Swept Light Sheet Microscopy (ASLM)^47^ can still struggle to acquire large volume sufficiently fast.

Similarly, there is a temporal imaging barrier (Fig. 2b). We currently cannot image rapid processes over long time periods due to the amount of data generated and the impact on sample health and bleaching of the fluorophores. For example, taking an image of 2048 × 2048 × 1 voxels every 1 ms over the period of one day amounts to a dataset of almost 700 TB. While data issues might be resolved in future with new hardware and larger storages, continuous imaging induces photo-toxic effects, which accumulate over imaging cycles^20,48^.

**Fig. 3:**
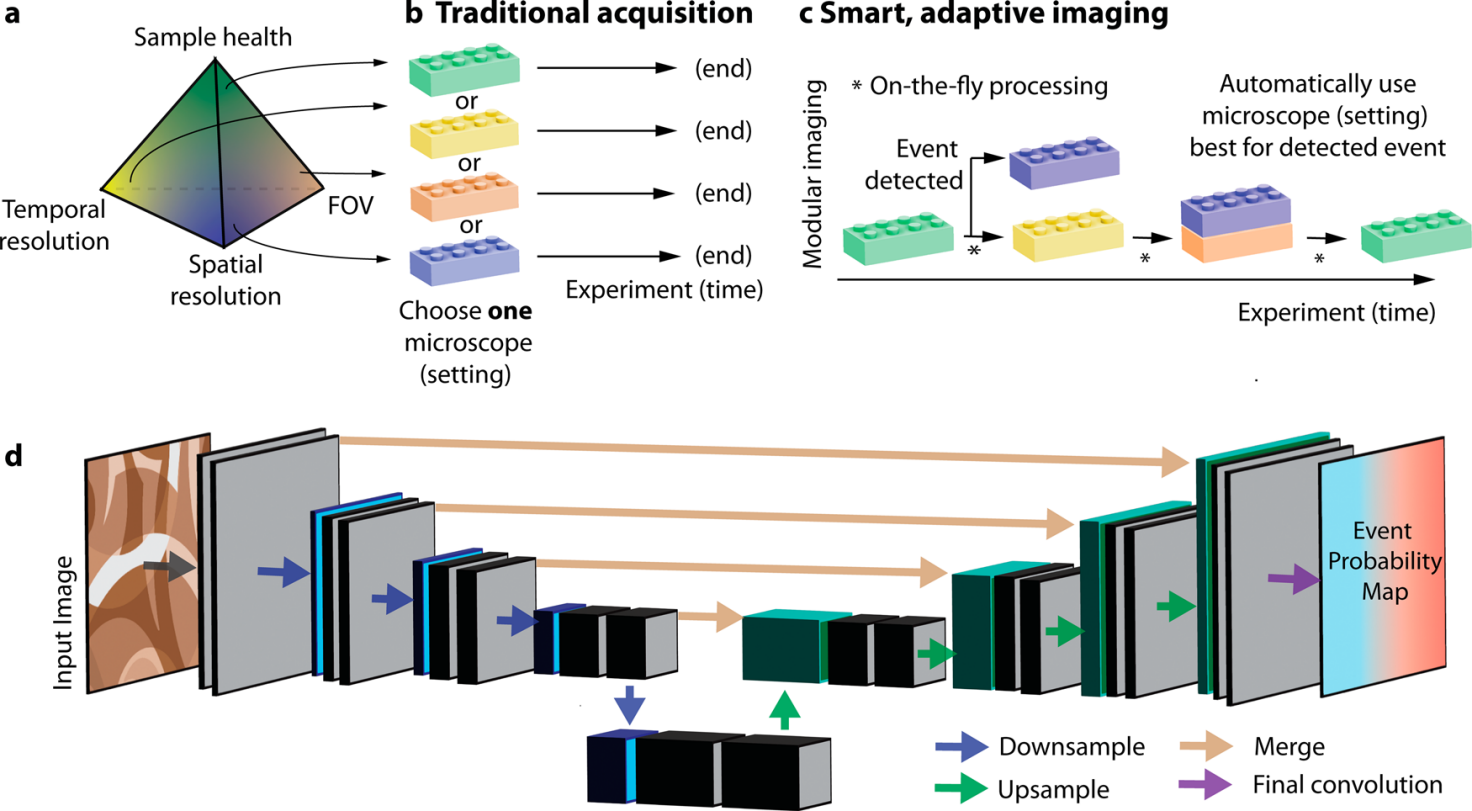
In the future, we expect novel smart and adaptive imaging schemes to overcome the traditional trade-offs and limitations of imaging. **a** Traditionally, an acquisition is governed by a limited photon budget of the sample. Therefore, improved spatial and temporal resolution is typically antagonistic to sample health and the field of view that is imaged. Optimizing one corner of the pyramid thus leads to trade-offs towards other corners. **b** Consequently, in a traditional acquisition *one* microscope and/or *one* microscope settings are chosen to best reflect the imaging needs defined by the biological questions asked: best sample health, e.g. through non-fluorescent acquisitions (bright-field imaging), highest spatial resolution, e.g. for studying molecular signaling (blue building block), largest field of view to e.g. capture a whole organism or organ such as the whole brain (orange building block), or highest temporal resolution to capture fast processes such as neuronal signaling or organismal movements (yellow building block). **c** In the future, we anticipate that new smart and adaptive imaging schemes will overcome the current limitation by providing modular imaging within one experiment. Thereby, a microscope will be able to use event-based detections to switch automatically between imaging modes, which optimize for example, large field of views (orange building block), spatial (blue building block) and temporal (yellow building blocks) resolution and sample health (green building blocks). **d** In a recent implementation of such a novel imaging scheme, Mahecic et al.^96^ utilized neural networks for event-based detections. Here, the architecture of the utilized U-Net network is displayed that takes an acquired input image and outputs an event-based probability map for guiding the microscope. The U-Net consists of encoder (downsampling layers, blue), decoder (upsampling, green) sections and connections between layers (beige).

Consequently, traditional acquisitions governed by Nyquist sampling are limited by trade-offs between sample health, temporal resolution, spatial resolution, and field of view or volumetric coverage, respectively (Fig. 3a). To account for these trade-offs, a microscopist has to choose one imaging modality to best fit the biological question at hand and perform an experiment with the chosen settings to the end (Fig. 3b).

## Methods to overcome scattering and absorption processes

To push the volumetric imaging barrier, multi-photon excitation^49–51^, wave-front shaping^52^, tissue clearing^53^ and expansion microscopy^54,55^ have been developed to overcome the physical limitations for imaging due to light-scattering and absorption processes. Physical scattering and absorption arises due to tissue-inherent absorbing chromophores such as blood, melanin, water or pigments, and small-and large-scale scatterers in the structure of cells and tissues^56,57^. This results in reduced penetration of optical microscopy into tissue, limiting imaging to few tens to hundreds of micrometers from the tissue surface^58^ (i.e. one optical mean free path).

Multi-photon excitation^49–51^ has increased the optical penetration depth to more than a mm in some tissues^49,59^. Importantly though, light-sheet microscopy does not benefit as strongly from multiphoton excitation than raster scanning techniques do. This is because a light-sheet microscope still needs to form a widefield image, which is severely limited by light-scattering in the visible wavelength. In contrast, multi-photon raster scanning microscopes do not need to form a sharp image with the returning fluorescence photons and as such can go much deeper. Thus, intravital light-sheet microscopy is currently limited to a depth of less than 100 microns in most tissues.

As an alternative, a judicious choice of a reasonably translucent model organism, such as zebrafish lines with no pigmentation^60^, has enabled light-sheet imaging in situ and in vivo. Furthermore, tuning the refractive index of the immersion medium better to the sample^61,62^ reduces scattering and thus improves penetration depth. Also, a shift to fluorescent probes in the near infrared II window (900–1700 nm) has shown promise to increase the reach of light-sheet microscopes in tissues^63^. Longer wavelengths have intrinsically a longer scattering mean free path, and can overlap with the absorption window of biological tissues^64^. Development of probes for this optical window, however, has remained challenging as absorbing and emitting at longer wavelengths necessitates increased electronic conjugation, which is often accompanied with reduced molecular rigidity, increased sources for non-radiative decay, and low quantum yields^65,66^. Quantum dots^67^ and carbon nanotubes^68^ have been used as alternative to fluorescent proteins/dye molecules, but complicate labeling specificity and biocompatibility. Therefore, the future of near infrared light-sheet imaging strongly depends on future breakthroughs in probe development.

Additionally, progress in wavefront correction schemes, in particular multi-conjugate adaptive optics (MCAO)^69,70^, may increase the optical penetration depth further. MCAO addresses the issue that conventional adaptive optics can only correct a small area, the so called isoplanatic patch^71,72^. In tissues, this patch can be smaller than the field of view of the camera, negating the benefits of parallelized detection of light-sheet microscopy. By correcting different regions of tissue separately, MCAO has the potential to increase the isoplanatic patch size^69,70^ and may enable effective light-sheet imaging in tissues. Conventional adaptive optics for light-sheet microscopy has been demonstrated^73,74^, but the setup featured a high complexity. To rapidly correct spatially varying aberrations, dedicated wavefront sensors and deformable mirrors were employed both in the excitation and emission path of the light-sheet microscope. As such, it may seem at first far-fetched to add even more components for MCAO, making such a system overly complex. However, we envision that through machine learning, aberrations can be sensed without dedicated wavefront sensors^75,76^ significantly easing the equipment constraints. Further, instead of deformable mirrors, transmissive deformable waveplates have shown promise for wavefront correction^77^. In principle, such devices can be stacked in an image space of the microscope to perform MCAO, or a dedicated, integrated 3D wavefront shaping device might be devised.

For fixed tissues, sample preparation through tissue clearing^53^ can largely overcome the depth limitations associated with light-scattering. Particularly interesting in this context is expansion microscopy^54,55^, which can physically magnify a sample tenfold or larger^78,79^ (Fig. 1d). This effectively increases the resolving power of any microscope by the expansion factor, and thus enables light-sheet microscopes to reach resolution levels that where hitherto limited to super-resolution microscopy (Fig. 2a). Therefore, expansion microscopy is a way to overcome the volumetric imaging barrier by modifying the sample, with the caveat that the expansion process might not always preserve the ultrastructure and careful validation is needed^80^. The challenge is now to image the thousandfold larger volumes effectively, which will even test the most efficient volumetric light-sheet microscopes. As expansion microscopy progresses, we see further need to engineer novel light-sheet microscopes with ever larger field of view, larger cameras and working distances. Also, techniques that rapidly tile^81,82^ or scan^39^ the light-sheet to cover large field of views might become more necessary in this quest.

## Smart, adaptive imaging schemes

While it is possible to push the imaging barriers with novel developments, as light-sheet microscopy^3^ and expansion microscopy^54,55^ have done, a complementary approach is to modularly combine the strengths of different techniques into one imaging workflow (Fig. 3c). Thereby, the microscope system will determine by itself when and how to apply which module, such as spatiotemporal sampling, field of view and sample irradiation. Thus, we envision that such smart and adaptive imaging schemes will overcome traditional Nyquist sampling and expand the capabilities of light microscopy, including light-sheet microscopy.

First steps towards such universal smart and adaptive schemes have already been achieved. An emerging requirement for any smart and adaptive imaging scheme is a feedback loop based on real-time, on-the-fly processing of the acquired data to monitor for changes.

Real-time analysis of microscope images has been established to improve imaging parameters within one imaging modality. In a landmark paper, McDole et al.^4^ applied adaptive light-sheet microscopy to capture mouse embryo development (Fig. 1b) by real-time specimen tracking and automatic adjustment of the overall imaging volume and other microscope parameters. The imaging scheme thereby compensates for drift, growth and changing optical properties, improving over the previously published automated microscopy routine AutoPilot^83^ that required near-constant size and shape. Important parameters for light-sheet based techniques are optimization of the imaging volume and spatial overlap between the light-sheet and detection focal planes including their relative offsets and angles^4,83]^. In our view, the main difference to a traditional microscope is the decoupling of illumination and detection, and hence this relative alignment is critical for best imaging performance. Moreover, imaging schemes have been devised to automatically find the best angles in SPIM acquisitions^31^ or tailor illumination dosage in super-resolution microscopy^84,85^ and multi-photon microscopy^86–88^. Additionally, automatic adjustment of the imaging volume to fit the sample morphology showed drastic reduction in the duration of imaging and overall light dose, and thus improved sample health^89–91^.

To change between imaging modalities (Fig. 3c), mechanisms to detect events of interest are required. Event-detection thereby relies on the early identification of changes in biological structures or behavior such as an upcoming cell division or cell signaling, to name a few examples. In an early implementation of event-driven microscopy, Almada et al.^92^ performed unsupervised, high-content, event-driven sample treatment and live-to-fixed imaging. Thereby, they relied on mitotic cell rounding as a biological cue, determined by on-the-fly cell segmentation with Otsu thresholding. Combining widefield imaging for event detection with STED super-resolution imaging, Alvelid et al.^93^ designed an automated multiscale method to selectively image protein recruitment, vesicle trafficking and biosensor activity with high-resolution. Applying GPU accelerated peak detection, they realized data processing on a millisecond time scale. Additionally, GPU-based deep learning networks such as the U-Net architecture^94^ (Fig. 3d) promise great potential for event-detection due to their inherently fast, parallel processing of large images once trained and the active development of specialized hardware, such as Tensor Processing Units (TPU)^95^. Mahecic et al.^96^ applied such a network for event detection of upcoming mitochondrial and bacterial divisions, enabling selective rapid imaging of these processes at rates matching their temporal dynamics.

While live imaging is currently the main driver of such smart acquisition schemes, we also envision them to become important in the exploration of cleared organs, and even entire animals. While time is not a hard barrier, after all the samples are no longer alive, it is still a factor. This is especially true for repeated experiments and particularly in clinical settings where mm-size biopsies are routine and cellular resolution needs to be achieved for accurate cell type identification for prognosis. The amount of data generated by imaging large, cleared tissues can also not be understated, especially in the context of expansion microscopy. Smart imaging schemes will therefore be crucial to explore cleared tissues and autonomously switch to higher resolution imaging only in areas of interest.

At the heart of smart and adaptive microscopes routines is the microscope control software that enables adaptive control schemes and event detections. In the available implementations, the importance of open-source control software has become evident. Open-source software allows for controlling and modifying every aspect of microscope acquisition and integration with available fast image analysis software. These efforts are spearheaded by open-source software such Micro-Manager^97^, Pycro-Manager^98^, AutoScanJ^99^, or other, Python-based control software^100,101^. As open-source software is often developed and maintained by few contributors, it will remain a challenge to maintain and adapt scripts to new hardware and incorporate new on-the-fly processing algorithms. Therefore, modularity of the software is essential, and containerization of image processing workflows could contribute to maintain compatibility, allowing for several software environments on one computer^102,103^. For commercial microscope providers, we believe it will be paramount to provide interfaces to these open-source tools. One way to achieve this could be through enabling network message triggered events in the acquisition protocols^99^.

While the fundamentals for smart and adaptive microscopes have been laid, the era of smart microscopes has just dawned. Recognizing the advancement that deep learning networks have achieved in other fields such as sequence-to-structure prediction with AlphaFold^104^ and large-scale generative language models with GPT^105^, we envision microscope experiments where a user could input keywords such as “capture all endocytosis events” and the microscope would then systematically image these events in the specimen.

This would require training deep learning networks with universal grounding in fundamental biology concepts and to associate biological terms with their visual microscopy appearances. The growing availability of public image repositories, e.g., the Image Data Resource^106^, and NIH’s recent guideline of making all image data associated with a publication available might be a first step towards this direction to train such networks. Challenges remain, for example, the availability of massive amounts of microscope data with insufficient annotation might render them less impactful to train the anticipated deep learning networks for microscopy. Crucially, unlike natural language, images are not bound to a standard visual ‘dictionary’ or ‘vocabulary’ but demonstrate significant visual heterogeneity even for the same biology. It remains an open question how to ensure generalizability and scalability of any trained networks beyond a single biological process and lab. Moreover, training of such a universal microscopy network will likely incur significant costs that are currently beyond the reach of microscope institutions, let alone individual labs. To overcome some of these limitations, self-supervising deep learning networks have shown promise in identifying similar morphologic features within large repositories of whole-slide histology images, independent of repository image size and with almost no annotations^107^. The development of techniques to learn from only weak or limited supervision^108^ or deploy expert-in-the-loop active learning^109^ to annotate and refine the ‘hard’ cases may also present a promising cost-efficient avenue to scale learning.

In a similar avenue, ‘unsupervised’ probabilistic models might learn the distribution of available images to enable the searching of rare events to uncover previously unknown biology. An example of such a rare find by human annotators has been the discovery of structures in zebrafish brain vasculature termed as Kugeln^110^ after many years of research and imaging of zebrafish brain vasculature. In future, a smart microscope might present such discoveries themselves.

Another current limitation for on-the-fly processing is the time required for data processing as a light-sheet microscope can easily generate gigabytes of data within seconds. However, we expect considerable progress towards faster processing pipelines in the foreseeable future. Besides progress in better algorithms, this acceleration will come in part by progress in computing hardware. Current computer architectures still predominantly rely on discrete, split CPU and GPU memory, which requires slow transfer of the data between them. In future, we expect that microscope acquisition will rely on single physical memory resource (e.g. Soc DRAM), shared by CPU and GPU, currently e.g. available on NVIDIA Jetson^111^, which will eliminate copying of data back and forth between CPU and GPU and thus make the best of both worlds available to fast processing – potentially even on the camera chip. Thereby, microscopy may benefit from development of tools that enable autonomous driving, where a huge number of images are analyzed on-the-fly to identify street hazards, other cars, or pedestrians.

## Alternative ways of overcoming sampling trade-offs in microscopy

In addition to changing the imaging parameters and modules during acquisition with smart and adaptive imaging schemes, acquisition schemes can be devised where a higher-resolution image data set is reconstructed after acquisition from a low-resolution scan or a scan which deliberately contains missing regions. This has the potential to significantly reduce the overall light dose on the sample, acquired data volume and acquisition time. After image reconstruction, the resolution of the original imaging system is recovered, or even increased, overcoming the traditional imaging trade-offs (Fig. 3a). With progress in the theory of image reconstruction with compressed sensing^112–114^ and machine learning^115–118^ approaches, we expect that such algorithms will become more widespread in the future.

Compressed sensing^112–114^ is a mathematical framework that describes how to capture and represent signals (images) at rates significantly below the Nyquist rate. The theory of compressed sensing is traditionally based on three concepts: sparsity, incoherence and random sampling^119^. If all three are given, successful reconstruction of an under-sampled signal can be achieved. A signal is sparse when it can be represented in a certain domain or basis with only few non-zero parameters. Therefore, the sparsity constraint is usually fulfilled for fluorescent microscopy as they are often already sparse in their pixel representation (e.g., few selectively labeled structures), or can be easily compressed, which means that there is a basis, e.g., in wavelets, in which many components are zero. However, incoherence (the values in the measurement matrix are uniformly spread out) and uniform random sampling are often lacking in microscopy^119^. After all, current image sensors acquire data deterministically over a 2D array, and not in a random fashion. Nevertheless, compressed sensing application have been demonstrated successfully and new principles for compressed sensing have been introduced to bridge the gap between theory and practice: asymptotic incoherence, asymptotic sparsity and multilevel sampling^119^.

Several successful applications of compressed sensing in imaging and microscopy have been demonstrated^120^. These applications include massively accelerated frame rate of cameras, reaching 100 billion frames per second^121^. Moreover, compressed sensing has been implemented on a lattice light-sheet microscope and an epifluorescence microscope to reduce light exposure and acquisition time 5–10 fold^122^, and applied for high-throughput anatomical imaging of whole mouse brains of ~400 mm^3^ on a timescale of ~10 min^123^. Importantly, compressed sensing reconstruction is unsupervised and does not require prior training data. Moreover, reconstruction accuracy improves as resolution increases^124^. This makes compressed sensing an appealing technique for the future of multi-resolution, smart light-sheet imaging schemes.

Similarly, deep learning networks can learn image restoration from training data^125–127^. Thereby, deep learning can address several limitations of compressed sensing^126^. Traditionally, compressed sensing requires a handcrafted reconstruction procedure, which might be difficult to establish for sophisticated image models. Moreover, such reconstruction procedures are based on iterative inverse optimization algorithms which tend to be slow and delicate to tune correctly, and thus reconstruction is hard to achieve in real-time. In contrast, reconstruction with deep learning requires only a single, fast forward propagation through the network. Moreover, the (asymptotic) incoherence of data required for compressed sensing is not strictly required for deep learning networks, in contrast, they might benefit from coherent data. Not surprisingly, applications of deep learning for image reconstructions is therefore a very active area of research and a variety of networks have been developed for this task^126,127^.

Deep learning, however, faces several challenges. Deep learning models do not yet provide the generalization, robustness and stability of reconstructions provided by established compressed sensing, and suffer from hallucinations, i.e. the creation of realistic looking artefacts^127,128^. This is related to the question of how well-trained networks generalize outside their training set and model fairness, the ability of models to equally capture and represent both common and rare phenotypes. We expect considerable progress in the next years to address these questions. The reporting of quantification of uncertainties will therefore be crucial to interpret reconstructions. To this end, models such as Bayesian inversion^129^ and techniques such as Bayesian dropout^130^ exist to generate uncertainty measures from deep learning models. However, the accuracy of the uncertainty measures require further external assessment to ascertain their ability to account for aleatoric and epistemic uncertainty^131,132^.

Moreover, the data-driven approach of deep learning networks depends on the availability of good (size, balance, and quality) training data reflective of the intended application. This is particularly true for image reconstruction, which requires an output with highest resolution data, in contrast to classification tasks and (binary) segmentations which are in essence coarse data. However, while popular networks such as GPT^105^ rely on an abundance of available data with limited restrictions, microscopy image reconstruction tasks are usually specialized applications with small datasets. One hope is that in the future, with mandates to host all microscope data accompanying a publication, vastly more and better annotated training data will eventually become available. Additionally, data augmentation, e.g., by geometric transformation such as image rotations, can increase the data size^133^. Moreover, application of a deep learning network that was pre-trained for different tasks on more diverse and larger datasets might be beneficial, a concept known as transfer learning^134^. Additionally, meta-learning approaches^135,136^ to specifically train networks in the few-image setting may yield more performant networks in real deployment. Similarly, more physically informed deep-learning network architectures, that model the image generation process, can help to ensure realistic predictions and reduce the number of free parameters to fit for faster, more generalizable learning^137,138^. Lastly, adopting a continuous learning paradigm instead of one-off training, may help to continuously adapt to new data and imaging conditions.

Despite these concerns, deep learning restoration techniques have been applied successfully and have even been granted approval by the FDA for select applications such as CT scans^139^. In super-resolution microscopy, considerable acquisition speed improvements have been reported through restoration^140–142^. For light-sheet microscopy, deep learning networks such as CARE^143^ have improved the SNR ratio of images acquired with less laser power or faster exposure. Interestingly, deep learning networks have also been applied to directly influence the sampling process. Horstmeyer *et al*. developed convolutional neural networks to optimize the physical layout of a microscope to improve accuracy of the identification of malaria-infected cells by 5–10%^144^. We expect that future developments will further leverage this avenue of co-optimizing image acquisition with deep learning networks for analysis. Understanding both the imaging system and process thereby will lead to faster processing times and better reconstructions^138^.

Ultimately, it is also important to realize that an image is often an intermediary step to the quantification of a biological process. Therefore, for many studies, a visually appealing deep-learning reconstructed image may be less important than having image data with rigorously quantifiable conclusions. This might alleviate some of the challenges described above but requires a precise understanding of the imaging system and image formation. Towards this end, Pégard et al. demonstrate compressive light-field microscopy, enabling real-time quantification of brain activity without ever reconstructing a 3D image^145^.

## Improving accessibility and usability of light-sheet microscopes

We anticipate that light-sheet technology itself will advance in the form of refined optical designs, better detectors, novel probes, NIR imaging capability and potentially non fluorescent contrast methods, such as Raman scattering. Some technical aspects of light-sheet technology may however been optimized to their maximum extent, such as the numerical aperture that can be covered in a light-sheet microscope^146,147^. Further gains in this area would likely also diminish the practicability of the instrument. This is imposed by the orthogonal configuration of LSFM and the fact that high NA objectives need a large opening angle. As a result, improving the lateral resolution beyond a certain threshold comes at the cost of reducing the axial resolution and vice versa, as the excitation and detection light cones share a limited solid angle.

Instead, we think that the future impact of light-sheet systems greatly depends on their practicality and applicability to biological and biomedical research questions. Many traditional light-sheet designs require non-traditional sample mounting^5,36^ and offer only limited space for the sample itself (Fig. 1a).

**Fig. 4:**
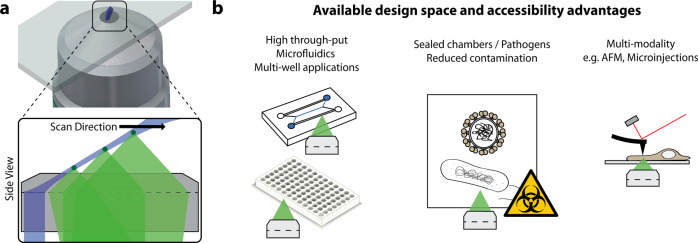
Novel engineering solutions for light-sheet microscopy provide improved accessibility to enable new applications and integration with other modalities. **a** Oblique Plane Light-Sheet Microscopy (OPM) is one example of open top geometries, where a high NA primary objective provides both, the illumination (blue) and detection (three fluorescent emitters along the light-sheet, shown in dark green, light green shows the fluorescence collected by OPM from these three emitters). This removes the need for additional illumination objectives as in traditional light-sheet microscopes, and thus provides novel available optical design space and improved accessibility. To scan a 3D volume, the light-sheet is scanned across the objective and no stage or sample movement is necessary. **b** OPM facilitates light-sheet microscopy for (i) novel high-throughput, multi-well applications, and microfluidics devices, (ii) imaging of new probes that are sealed to reduce contaminations over long time periods and imaging of pathogens such as bacteria and viruses, (iii) and combinations with other modalities such as Atomic Force Microscopy (AFM).

A promising alternative are open top^37,148^ and oblique plane microscopes (OPM)^8,28,149^, which leave one half space free (Fig. 4a) to place samples of, in principle, arbitrary sizes. Progress in the optical design of these microscopes has enabled microscopes with large millimeter sized field of views^150–154^ and microscopes with high-resolution^8,155^, and even with both modalities^156^. Recently, also commercial microscopes have been developed on the basis of open-top microscopy^157^. As such, we believe that open top and OPM systems will enable a widespread adoption of light-sheet microscopy, as conventional sample mounting methods can be employed, and integration in standard microscope bodies is in principle possible with OPM. This opens the way for three dimensional high-throughput imaging using multi-well plates, imaging of toxic or infectious specimens contained in (sealed) dishes, and multimodal imaging approaches (Fig. 4b).

Open top and OPM have also opened new design spaces for optical engineering. OPM relies on remote refocusing^158^, which describes the ability to create an aberration-free 3D image of the specimen in a remote space away from the sample. The tilted light-sheet plane within that 3D image can then be mapped with another microscope onto a camera. While the remote focusing principle^158^ underlying OPM has been established over a decade ago, it has been recently re-analyzed^159^ to enable imaging across different refractive indices. The new findings may enable high resolution light-sheet imaging in any immersion media, furthering the versatility and applicability of light-sheet microscopy. This should serve just as one example on how improvements might still come from discoveries of optical principles and theory, as well as engineering.

## Conclusion

We expect that light-sheet microscopy will play an important role in the biomedical sciences and clinical applications for microscopic and macroscopic imaging in the future. Its combination of rapid yet gentle volumetric imaging will serve as the basis of physiologically relevant studies of cellular biology in cell culture, in organoids, in (engineered) tissue, clinical biopsies, and in entire animals. As such, one can dream big, a future where sub-cellular biology can be studied live in its native context, without the limitations imposed by traditional cell culture methods on coverslips.

To achieve this dream, we expect light-sheet microscopes to overcome the volumetric and temporal imaging barriers imposed by constrains of the microscope systems and sample. This will no longer be a task that a human microscope operator, or image analyst will be able to handle alone. Instead, novel smart and adaptive microscope control schemes will explore samples in an autonomous, self-driving fashion to image processes of interest selectively at rates matching their dynamics. These schemes will enable new autonomous biological discoveries and systematic imaging studies of processes that take place over multiple length and timescales.

Besides increased throughput, such acquisition schemes also promise to rein in the data deluge. Current light-sheet data can already reach petabyte scales and will likely reach even higher orders of magnitudes soon. As smart and adaptive data acquisition schemes no longer follow Nyquist sampling on the finest level across the entirety of the data set, finest sampling will only be applied selectively. Moreover, algorithmic selection of regions of interests will remove human bias and thus improve reproducibility of imaging studies.

As with any look into the future, it is likely that the field could take very different directions. After all, who would have foreseen expansion microscopy^54,55^ before 2015, which has impacted fluorescence microscopy in unimaginable ways. Thus, while we are excited about the possibilities that we have described herein, we also hope that the microscope community will remain as imaginative as it has been over the last 20 years, holding many more surprises in store.

### Reporting summary

Further information on research design is available in the Nature Portfolio Reporting Summary linked to this article.

## Supplementary information

Reporting Summary
